# You can run but you can't hide: tracking T cells in metastatic melanoma patients treated with tumor-infiltrating lymphocytes

**DOI:** 10.1186/2051-1426-3-S2-P22

**Published:** 2015-11-04

**Authors:** Cara Haymaker, Marie-Andrée Forget, Caitlin Creasy, Krit Ritthipichai, Patrick Hwu, Chantale Bernatchez

**Affiliations:** 1 UT MD Anderson Cancer Center, Houston, TX, USA

## Background

Adoptive T cell therapy using tumor-infiltrating lymphocytes (TIL) for patients with metastatic melanoma has been shown to have a 45-50% response rate as demonstrated by multiple institutions worldwide. However, understanding how long infused T cells persist in patients remains unknown and the specificity of the majority of the T cells is unidentified.

## Methods

In this study, we have utilized high-throughput CDR3 sequencing of the T cell receptor, thus providing a unique genetic signature for each individual T cell since it is maintained throughout division and present on the respective daughter cells. To this end, the TIL infusion product from 14 treated melanoma patients (7 CR/PR and 7 SD/PD) were sorted into CD8 and CD4 subsets and sequenced to provide a signature for the infused cells. Blood was collected at regular intervals following infusion and the CDR3s present in the PBMC pool were analyzed for the presence of the infused TIL subsets. In addition, diversity level of the infusion product as well as the lymphocyte pool reconstituting the patient following lymphodepletion was assessed. Flow cytometric analysis was also performed on each infusion product.

## Results

Interestingly, long-term responding patients were found to have TIL present in their PBMCs even 4.5 years following infusion. The percentage of infused TIL persisting in the blood of responding patients varied between 10-20% of the circulating T cells (as determined by CDR3 sequencing). Assessment of the diversity of the CD8^+^ subset of the infusion product showed that the TIL from responding patients were more oligoclonal than TIL from non-responding patients. In addition, the diversity of the lymphocyte pool reconstituting in responding patients did not re-attain the same level of diversity as the baseline pool prior to TIL infusion demonstrating a lasting impact on the immune system by this therapy.

## Conclusions

Overall, this study demonstrates that TIL are capable of persisting long-term in responding patients and that the diversity of the CD8^+^ T cells within the infusion product may correlate with clinical response. In addition, this technology allows us to determine, for the first time, what type of memory pool develops from these infused cells that persist and whether infused TIL traffic to emerging tumor sites.

